# Supplementary material for: An assessment of the impacts of litter treatments on the litter quality and broiler performance: A systematic review and meta-analysis
Source: PLoS One. 2020 May 6;15(5):e0232853. doi: 10.1371/journal.pone.0232853 (PMC7202646; doi:10.1371/journal.pone.0232853)
Supplement: S1 Table — (DOCX) [file pone.0232853.s001.docx]

S1_Table. Data for Feed intake meta-analysis

| Study name | Treated Group N | Treated Group mean | Treated Group Standard deviation | Control Group N | Control Group mean | Control Group Standard deviation | Treatment |
| --- | --- | --- | --- | --- | --- | --- | --- |
| Avcilar et al. 2018a | 6 | 3328.0 | 22.011 | 6 | 3310.0 | 22.011 | Adsorber |
| Avcilar et al. 2018b | 6 | 3317.0 | 22.011 | 6 | 3310.0 | 22.011 | Adsorber |
| Bruno et al. 1999a | 4 | 4833.0 | 232.467 | 4 | 4954.0 | 238.287 | Gypsum |
| Bruno et al. 1999b | 4 | 4986.0 | 239.827 | 4 | 4954.0 | 238.287 | Gypsum |
| Bruno et al. 1999c | 4 | 4985.0 | 239.779 | 4 | 4954.0 | 238.287 | Gypsum |
| Bruno et al. 1999d | 4 | 5111.0 | 245.839 | 4 | 4954.0 | 238.287 | Gypsum |
| Ferreira et al. 2004a | 4 | 4.082 | 0.265 | 4 | 4.193 | 0.272 | Acidifying |
| Ferreira et al. 2004b | 4 | 3.885 | 0.252 | 4 | 4.193 | 0.272 | Gypsum |
| Ferreira et al. 2004d | 4 | 4.195 | 0.272 | 4 | 4.193 | 0.272 | Alkalizing |
| Furlan, 2017a | 7 | 5207.0 | 64.710 | 7 | 5163.0 | 64.710 | Acidifying |
| Furlan, 2017b | 7 | 5248.0 | 64.710 | 7 | 5163.0 | 64.710 | Acidifying |
| Furlan, 2017c | 7 | 5228.0 | 64.710 | 7 | 5163.0 | 64.710 | Acidifying |
| Furlan, 2017d | 7 | 5231.0 | 64.710 | 7 | 5185.0 | 64.710 | Acidifying |
| Furlan, 2017e | 7 | 5131.0 | 64.710 | 7 | 5185.0 | 64.710 | Acidifying |
| Furlan, 2017f | 7 | 5188.0 | 64.710 | 7 | 5185.0 | 64.710 | Acidifying |
| Furlan, 2017g | 7 | 5176.0 | 99.890 | 7 | 5197.0 | 99.890 | Acidifying |
| Garrido et al. 2004 | 6 | 338.90 | 14.000 | 6 | 327.6 | 4.100 | Acidifying |
| Oliveira et al. 2015b | 4 | 4784.0 | 274.602 | 4 | 4947.0 | 283.958 | Acidifying |
| Oliveira et al. 2015c | 4 | 4914.0 | 282.064 | 4 | 4947.0 | 283.958 | Gypsum |
| Oliveira et al. 2015d | 4 | 5026.0 | 288.492 | 4 | 4947.0 | 283.958 | Alkalizing |
| Oliveira et al. 2015e | 4 | 4863.0 | 279.136 | 4 | 4947.0 | 283.958 | Alkalizing |
| Oliveira et al. 2015f | 4 | 5103.0 | 292.912 | 4 | 4947.0 | 283.958 | Adsorber |
| Oliveira et al. 2015g | 4 | 4845.0 | 278.103 | 4 | 4947.0 | 283.958 | Adsorber |
| Purswell et al. 2013a | 12 | 7285.0 | 142.028 | 12 | 7362.0 | 142.028 | Acidifying |
| Purswell et al. 2013b | 12 | 7271.0 | 142.028 | 12 | 7362.0 | 142.028 | Acidifying |
| Purswell et al. 2013c | 12 | 7285.0 | 142.028 | 12 | 7362.0 | 142.028 | Acidifying |
| Purswell et al. 2013d | 12 | 7357.0 | 142.028 | 12 | 7362.0 | 142.028 | Acidifying |
| Ruiz et al. 2008b | 4 | 4168.6 | 95.920 | 4 | 4008.1 | 95.920 | Alkalizing |
| Ruiz et al. 2008c | 4 | 4033.4 | 95.920 | 4 | 4008.1 | 95.920 | Alkalizing |
| Sahoo et al. 2017a | 3 | 3655.1 | 40.824 | 3 | 3713.8 | 70.512 | Acidifying |
| Sahoo et al. 2017b | 3 | 3688.6 | 62.614 | 3 | 3713.8 | 70.512 | Acidifying |
| Zhang et al., 2011a | 3 | 81.2 | 2.771 | 3 | 81.2 | 1.559 | Acidifying |
| Zhang et al., 2011b | 3 | 79.3 | 1.039 | 3 | 79.3 | 5.543 | Acidifying |
| Zhang et al., 2011c | 3 | 79.4 | 4.503 | 3 | 83.2 | 1.559 | Acidifying |
| Taherparvar et al. 2016a | 3 | 115.2 | 2.910 | 3 | 116.9 | 2.910 | Adsorber |
| Taherparvar et al. 2016b | 3 | 115.6 | 2.910 | 3 | 116.9 | 2.910 | Alkalizing |
