# Supplementary material for: An assessment of the impacts of litter treatments on the litter quality and broiler performance: A systematic review and meta-analysis
Source: PLoS One. 2020 May 6;15(5):e0232853. doi: 10.1371/journal.pone.0232853 (PMC7202646; doi:10.1371/journal.pone.0232853)
Supplement: S2 Table — (DOCX) [file pone.0232853.s002.docx]

S2_Table. Data for weight gain meta-analysis

| Study name | Treated Group N | Treated Group mean | Treated Group Standard deviation | Control Group N | Control Group mean | Control Group Standard deviation | Treatment |
| --- | --- | --- | --- | --- | --- | --- | --- |
| Avcilar et al. 2018a | 6 | 1987.0 | 25.080 | 6 | 1988.0 | 25.080 | Adsorber |
| Avcilar et al. 2018b | 6 | 2001.0 | 25.080 | 6 | 1988.0 | 25.080 | Adsorber |
| Bruno et al. 1999a | 4 | 2412.0 | 101.786 | 4 | 2402.0 | 101.364 | Gypsum |
| Bruno et al. 1999b | 4 | 2549.0 | 107.568 | 4 | 2402.0 | 101.364 | Gypsum |
| Bruno et al. 1999c | 4 | 2539.0 | 107.146 | 4 | 2402.0 | 101.364 | Gypsum |
| Bruno et al. 1999d | 4 | 2539.0 | 107.146 | 4 | 2402.0 | 101.364 | Gypsum |
| Ferreira et al. 2004a | 4 | 2.369 | 0.080 | 4 | 2.396 | 0.081 | Acidifying |
| Ferreira et al. 2004b | 4 | 2.294 | 0.078 | 4 | 2.396 | 0.081 | Gypsum |
| Ferreira et al. 2004d | 4 | 2.358 | 0.080 | 4 | 2.396 | 0.081 | Alkalizing |
| Furlan, 2017a | 7 | 3384.0 | 124.821 | 7 | 3213.0 | 124.821 | Acidifying |
| Furlan, 2017b | 7 | 3211.0 | 124.821 | 7 | 3213.0 | 124.821 | Acidifying |
| Furlan, 2017c | 7 | 3414.0 | 124.821 | 7 | 3213.0 | 124.821 | Acidifying |
| Furlan, 2017d | 7 | 2888.0 | 124.821 | 7 | 3054.0 | 124.821 | Acidifying |
| Furlan, 2017e | 7 | 3125.0 | 124.821 | 7 | 3054.0 | 124.821 | Acidifying |
| Furlan, 2017f | 7 | 3107.0 | 124.821 | 7 | 3054.0 | 124.821 | Acidifying |
| Furlan, 2017g | 7 | 3017.0 | 59.482 | 7 | 3047.0 | 59.482 | Acidifying |
| Mcward and Taylor 2000a | 10 | 2.744 | 0.060 | 10 | 2.606 | 0.128 | Acidifying |
| Mcward and Taylor 2000b | 10 | 2.665 | 0.077 | 10 | 2.606 | 0.128 | Acidifying |
| Mcward and Taylor 2000c | 10 | 2.704 | 0.067 | 10 | 2.606 | 0.128 | Acidifying |
| Mcward and Taylor 2000d | 10 | 2.649 | 0.042 | 10 | 2.434 | 0.105 | Acidifying |
| Mcward and Taylor 2000e | 10 | 2.666 | 0.030 | 10 | 2.434 | 0.105 | Acidifying |
| Mcward and Taylor 2000f | 10 | 2.644 | 0.058 | 10 | 2.434 | 0.105 | Acidifying |
| Oliveira et al. 2015b | 4 | 2625.0 | 106.575 | 4 | 2663.0 | 108.118 | Acidifying |
| Oliveira et al. 2015c | 4 | 2573.0 | 104.464 | 4 | 2663.0 | 108.118 | Gypsum |
| Oliveira et al. 2015d | 4 | 2564.0 | 104.098 | 4 | 2663.0 | 108.118 | Alkalizing |
| Oliveira et al. 2015e | 4 | 2608.0 | 105.885 | 4 | 2663.0 | 108.118 | Alkalizing |
| Oliveira et al. 2015f | 4 | 2651.0 | 107.631 | 4 | 2663.0 | 108.118 | Adsorber |
| Oliveira et al. 2015g | 4 | 2677.0 | 108.686 | 4 | 2663.0 | 108.118 | Adsorber |
| Purswell et al. 2013a | 12 | 3915.0 | 110.851 | 12 | 3883.0 | 110.851 | Acidifying |
| Purswell et al. 2013b | 12 | 3851.0 | 110.851 | 12 | 3883.0 | 110.851 | Acidifying |
| Purswell et al. 2013c | 12 | 3869.0 | 110.851 | 12 | 3883.0 | 110.851 | Acidifying |
| Purswell et al. 2013d | 12 | 3919.0 | 110.851 | 12 | 3883.0 | 110.851 | Acidifying |
| Sahoo et al. 2017a | 3 | 1813.0 | 3.377 | 3 | 1770.06 | 10.548 | Acidifying |
| Sahoo et al. 2017b | 3 | 1860.11 | 8.054 | 3 | 1770.06 | 10.548 | Acidifying |
| Zhang et al., 2011a | 3 | 39.7 | 0.693 | 3 | 41.1 | 0.866 | Acidifying |
| Zhang et al., 2011b | 3 | 37.0 | 3.118 | 3 | 39.3 | 1.386 | Acidifying |
| Zhang et al., 2011c | 3 | 38.0 | 0.520 | 3 | 39.7 | 1.039 | Acidifying |
| Taherparvar et al. 2016a | 3 | 68.25 | 2.685 | 3 | 68.06 | 2.685 | Adsorber |
| Taherparvar et al. 2016b | 3 | 67.59 | 2.685 | 3 | 68.06 | 2.685 | Alkalizing |
