# Supplementary material for: An assessment of the impacts of litter treatments on the litter quality and broiler performance: A systematic review and meta-analysis
Source: PLoS One. 2020 May 6;15(5):e0232853. doi: 10.1371/journal.pone.0232853 (PMC7202646; doi:10.1371/journal.pone.0232853)
Supplement: S3 Table — (DOCX) [file pone.0232853.s003.docx]

S3_Table. Data for feed conversion meta-analysis.

| Study name | Treated Group N | Treated Group mean | Treated Group Standard deviation | Control Group N | Control Group mean | Control Group Standard deviation | Treatment |
| --- | --- | --- | --- | --- | --- | --- | --- |
| Avcilar et al. 2018a | 6 | 1.680 | 0.020 | 6 | 1.670 | 0.020 | Adsorber |
| Avcilar et al. 2018b | 6 | 1.660 | 0.020 | 6 | 1.670 | 0.020 | Adsorber |
| Bruno et al. 1999a | 4 | 2.000 | 0.089 | 4 | 2.060 | 0.091 | Gypsum |
| Bruno et al. 1999b | 4 | 1.950 | 0.087 | 4 | 2.060 | 0.091 | Gypsum |
| Bruno et al. 1999c | 4 | 1.960 | 0.087 | 4 | 2.060 | 0.091 | Gypsum |
| Bruno et al. 1999d | 4 | 2.010 | 0.089 | 4 | 2.060 | 0.091 | Gypsum |
| Ferreira et al. 2004a | 4 | 1.760 | 0.097 | 4 | 1.780 | 0.098 | Acidifying |
| Ferreira et al. 2004b | 4 | 1.740 | 0.096 | 4 | 1.780 | 0.098 | Gypsum |
| Ferreira et al. 2004d | 4 | 1.810 | 0.100 | 4 | 1.780 | 0.098 | Alkalizing |
| Furlan, 2017a | 7 | 1.538 | 0.064 | 7 | 1.606 | 0.064 | Acidifying |
| Furlan, 2017b | 7 | 1.634 | 0.064 | 7 | 1.606 | 0.064 | Acidifying |
| Furlan, 2017c | 7 | 1.531 | 0.064 | 7 | 1.606 | 0.064 | Acidifying |
| Furlan, 2017d | 7 | 1.811 | 0.064 | 7 | 1.697 | 0.064 | Acidifying |
| Furlan, 2017e | 7 | 1.641 | 0.064 | 7 | 1.697 | 0.064 | Acidifying |
| Furlan, 2017f | 7 | 1.669 | 0.064 | 7 | 1.697 | 0.064 | Acidifying |
| Furlan, 2017g | 7 | 1.720 | 0.024 | 7 | 1.710 | 0.024 | Acidifying |
| Garrido et al. 2004 | 6 | 1.820 | 0.090 | 6 | 1.780 | 0.030 | Acidifying |
| Li et al., 2013 | 3 | 1.880 | 0.104 | 3 | 1.930 | 0.052 | Acidifying |
| Mcward and Taylor 2000a | 10 | 1.836 | 0.034 | 10 | 1.819 | 0.018 | Acidifying |
| Mcward and Taylor 2000b | 10 | 1.846 | 0.035 | 10 | 1.819 | 0.018 | Acidifying |
| Mcward and Taylor 2000c | 10 | 1.839 | 0.024 | 10 | 1.819 | 0.018 | Acidifying |
| Mcward and Taylor 2000d | 10 | 1.784 | 0.016 | 10 | 1.843 | 0.032 | Acidifying |
| Mcward and Taylor 2000e | 10 | 1.752 | 0.018 | 10 | 1.843 | 0.032 | Acidifying |
| Mcward and Taylor 2000f | 10 | 1.774 | 0.030 | 10 | 1.843 | 0.032 | Acidifying |
| Nagaraj et al. 2007a | 4 | 1.867 | 0.052 | 4 | 1.872 | 0.052 | Acidifying |
| Nagaraj et al. 2007b | 4 | 1.869 | 0.052 | 4 | 1.872 | 0.052 | Acidifying |
| Nagaraj et al. 2007c | 4 | 1.881 | 0.052 | 4 | 1.872 | 0.052 | Acidifying |
| Oliveira et al. 2015b | 4 | 1.830 | 0.137 | 4 | 1.850 | 0.138 | Acidifying |
| Oliveira et al. 2015c | 4 | 1.910 | 0.143 | 4 | 1.850 | 0.138 | Gypsum |
| Oliveira et al. 2015d | 4 | 1.950 | 0.146 | 4 | 1.850 | 0.138 | Alkalizing |
| Oliveira et al. 2015e | 4 | 1.860 | 0.139 | 4 | 1.850 | 0.138 | Alkalizing |
| Oliveira et al. 2015f | 4 | 1.920 | 0.144 | 4 | 1.850 | 0.138 | Adsorber |
| Oliveira et al. 2015g | 4 | 1.810 | 0.135 | 4 | 1.850 | 0.138 | Adsorber |
| Purswell et al. 2013a | 12 | 1.860 | 0.035 | 12 | 1.880 | 0.035 | Acidifying |
| Purswell et al. 2013b | 12 | 1.870 | 0.035 | 12 | 1.880 | 0.035 | Acidifying |
| Purswell et al. 2013c | 12 | 1.870 | 0.035 | 12 | 1.880 | 0.035 | Acidifying |
| Purswell et al. 2013d | 12 | 1.860 | 0.035 | 12 | 1.880 | 0.035 | Acidifying |
| Ruiz et al. 2008b | 4 | 1.890 | 0.020 | 4 | 1.850 | 0.020 | Alkalizing |
| Ruiz et al. 2008c | 4 | 1.880 | 0.020 | 4 | 1.850 | 0.020 | Alkalizing |
| Sahoo et al. 2017a | 3 | 2.020 | 0.035 | 3 | 2.100 | 0.017 | Acidifying |
| Sahoo et al. 2017b | 3 | 1.980 | 0.035 | 3 | 2.100 | 0.017 | Acidifying |
| Zhang et al., 2011a | 3 | 2.050 | 0.052 | 3 | 1.980 | 0.035 | Acidifying |
| Zhang et al., 2011b | 3 | 2.150 | 0.208 | 3 | 2.020 | 0.087 | Acidifying |
| Zhang et al., 2011c | 3 | 2.090 | 0.087 | 3 | 2.090 | 0.069 | Acidifying |
| Taherparvar et al. 2016a | 3 | 1.630 | 0.069 | 3 | 1.610 | 0.069 | Adsorber |
| Taherparvar et al. 2016b | 3 | 1.590 | 0.069 | 3 | 1.610 | 0.069 | Alkalizing |
