# Supplementary material for: An assessment of the impacts of litter treatments on the litter quality and broiler performance: A systematic review and meta-analysis
Source: PLoS One. 2020 May 6;15(5):e0232853. doi: 10.1371/journal.pone.0232853 (PMC7202646; doi:10.1371/journal.pone.0232853)
Supplement: S4 Table — (DOCX) [file pone.0232853.s004.docx]

S4_Table. Data for mortality rate meta-analysis.

| Study name | Treated Group N | Treated Group mean | Treated Group Standard deviation | Control Group N | Control Group mean | Control Group Standard deviation | Treatment |
| --- | --- | --- | --- | --- | --- | --- | --- |
| Bruno et al. 1999a | 4 | 11.140 | 4.885 | 4 | 12.510 | 5.486 | Gypsum |
| Bruno et al. 1999b | 4 | 12.090 | 5.301 | 4 | 12.510 | 5.486 | Gypsum |
| Bruno et al. 1999c | 4 | 10.570 | 4.635 | 4 | 12.510 | 5.486 | Gypsum |
| Bruno et al. 1999d | 4 | 10.520 | 4.613 | 4 | 12.510 | 5.486 | Gypsum |
| Li et al., 2013 | 3 | 4.170 | 0.641 | 3 | 4.790 | 0.849 | Acidifying |
| Nagaraj et al. 2007a | 4 | 1.340 | 1.920 | 4 | 2.680 | 1.920 | Acidifying |
| Nagaraj et al. 2007b | 4 | 0.460 | 1.920 | 4 | 2.680 | 1.920 | Acidifying |
| Nagaraj et al. 2007c | 4 | 2.710 | 1.920 | 4 | 2.680 | 1.920 | Acidifying |
| Purswell et al. 2013a | 12 | 0.400 | 0.346 | 12 | 1.500 | 0.346 | Acidifying |
| Purswell et al. 2013b | 12 | 1.100 | 0.346 | 12 | 1.500 | 0.346 | Acidifying |
| Purswell et al. 2013c | 12 | 1.000 | 0.346 | 12 | 1.500 | 0.346 | Acidifying |
| Purswell et al. 2013d | 12 | 0.900 | 0.346 | 12 | 1.500 | 0.346 | Acidifying |
| Ruiz et al. 2008b | 4 | 5.990 | 0.380 | 4 | 4.970 | 0.380 | Alkalizing |
| Ruiz et al. 2008c | 4 | 5.990 | 0.380 | 4 | 4.970 | 0.380 | Alkalizing |
