# Supplementary material for: An assessment of the impacts of litter treatments on the litter quality and broiler performance: A systematic review and meta-analysis
Source: PLoS One. 2020 May 6;15(5):e0232853. doi: 10.1371/journal.pone.0232853 (PMC7202646; doi:10.1371/journal.pone.0232853)
Supplement: S5 Table — (DOCX) [file pone.0232853.s005.docx]

S5_Table. Data for ammonia concentration meta-analysis.

| Study name | Treated Group N | Treated Group mean | Treated Group Standard deviation | Control Group N | Control Group mean | Control Group Standard deviation | Treatment |
| --- | --- | --- | --- | --- | --- | --- | --- |
| Chung et al. 2015 | 4 | 15.700 | 1.120 | 4 | 23.060 | 1.120 | Acidifying |
| Loch et al. 2011b | 4 | 95.390 | 14.757 | 4 | 87.340 | 13.511 | Acidifying |
| Loch et al. 2011c | 4 | 153.820 | 23.796 | 4 | 87.340 | 13.511 | Gypsum |
| Loch et al. 2011d | 4 | 117.090 | 18.114 | 4 | 87.340 | 13.511 | Alkalizing |
| Loch et al. 2011e | 4 | 119.970 | 18.559 | 4 | 87.340 | 13.511 | Alkalizing |
| Loch et al. 2011f | 4 | 112.430 | 17.393 | 4 | 87.340 | 13.511 | Adsorber |
| Loch et al. 2011g | 4 | 205.950 | 31.860 | 4 | 87.340 | 13.511 | Adsorber |
| Madrid et al., 2012 | 2 | 4.800 | 0.800 | 2 | 10.900 | 0.900 | Acidifying |
| Purswell et al. 2013a | 12 | 290.100 | 51.269 | 12 | 255.800 | 51.269 | Acidifying |
| Purswell et al. 2013b | 12 | 243.300 | 51.269 | 12 | 255.800 | 51.269 | Acidifying |
| Purswell et al. 2013c | 12 | 199.900 | 51.269 | 12 | 255.800 | 51.269 | Acidifying |
| Purswell et al. 2013d | 12 | 232.70 | 51.269 | 12 | 255.800 | 51.269 | Acidifying |
| Avcilar et al. 2018a | 6 | 3.000 | 0.113 | 6 | 3.320 | 0.113 | Adsorber |
| Avcilar et al. 2018b | 6 | 2.880 | 0.113 | 6 | 3.320 | 0.113 | Adsorber |
| Bordignon 2013a | 4 | 0.410 | 0.099 | 4 | 1.370 | 0.331 | Gypsum |
| Bordignon 2013b | 4 | 0.970 | 0.234 | 4 | 1.370 | 0.331 | Alkalizing |
| Bordignon 2013c | 4 | 0.280 | 0.068 | 4 | 1.370 | 0.331 | Superphosphate |
| Bordignon 2013d | 4 | 0.040 | 0.010 | 4 | 1.370 | 0.331 | Acidifying |
| Bordignon 2013e | 4 | 1.020 | 0.246 | 4 | 1.370 | 0.331 | Alkalizing |
| Sampaio et al. 1999a | 4 | 7.300 | 0.030 | 4 | 8.700 | 0.036 | Gypsum |
| Sampaio et al. 1999b | 4 | 5.570 | 0.023 | 4 | 8.700 | 0.036 | Gypsum |
| Sampaio et al. 1999c | 4 | 5.330 | 0.022 | 4 | 8.700 | 0.036 | Gypsum |
| Sampaio et al. 1999d | 4 | 5.180 | 0.021 | 4 | 8.700 | 0.036 | Gypsum |
| Furlan, 2017a | 7 | 2.430 | 0.272 | 7 | 2.730 | 0.272 | Acidifying |
| Furlan, 2017b | 7 | 1.920 | 0.272 | 7 | 2.730 | 0.272 | Acidifying |
| Furlan, 2017c | 7 | 2.930 | 0.272 | 7 | 2.730 | 0.272 | Acidifying |
| Furlan, 2017d | 7 | 3.260 | 0.272 | 7 | 2.940 | 0.272 | Acidifying |
| Furlan, 2017e | 7 | 3.420 | 0.272 | 7 | 2.940 | 0.272 | Acidifying |
| Furlan, 2017f | 7 | 3.460 | 0.272 | 7 | 2.940 | 0.272 | Acidifying |
| Furlan, 2017g | 7 | 1.080 | 0.072 | 7 | 1.980 | 0.072 | Acidifying |
| Oliveira et al. 2003a | 4 | 32.060 | 8.050 | 4 | 83.820 | 21.047 | Acidifying |
| Oliveira et al. 2003b | 4 | 11.290 | 2.835 | 4 | 83.820 | 21.047 | Gypsum |
| Oliveira et al. 2003c | 4 | 78.100 | 19.611 | 4 | 83.820 | 21.047 | Alkalizing |
| Oliveira et al. 2004a | 4 | 3.140 | 0.582 | 4 | 6.940 | 1.287 | Acidifying |
| Oliveira et al. 2004b | 4 | 5.760 | 1.068 | 4 | 6.940 | 1.287 | Gypsum |
| Oliveira et al. 2004c | 4 | 6.220 | 1.154 | 4 | 6.940 | 1.287 | Superphosphate |
| Oliveira et al. 2004d | 4 | 7.610 | 1.412 | 4 | 6.940 | 1.287 | Alkalizing |
| Li et al., 2013 | 3 | 0.680 | 0.242 | 3 | 0.760 | 0.052 | Acidifying |
