# Supplementary material for: An assessment of the impacts of litter treatments on the litter quality and broiler performance: A systematic review and meta-analysis
Source: PLoS One. 2020 May 6;15(5):e0232853. doi: 10.1371/journal.pone.0232853 (PMC7202646; doi:10.1371/journal.pone.0232853)
Supplement: S6 Table — (DOCX) [file pone.0232853.s006.docx]

S6_Table. Data for pH meta-analysis.

| Study name | Treated Group N | Treated Group mean | Treated Group Standard deviation | Control Group N | Control Group mean | Control Group Standard deviation | Treatment |
| --- | --- | --- | --- | --- | --- | --- | --- |
| Avcilar et al. 2018a | 6 | 8.10 | 0.083 | 6 | 8.20 | 0.083 | Adsorber |
| Avcilar et al. 2018b | 6 | 7.90 | 0.083 | 6 | 8.20 | 0.083 | Adsorber |
| Bordignon 2013a | 4 | 8.40 | 0.459 | 4 | 7.64 | 0.417 | Gypsum |
| Bordignon 2013b | 4 | 7.58 | 0.414 | 4 | 7.64 | 0.417 | Alkalizing |
| Bordignon 2013c | 4 | 6.70 | 0.366 | 4 | 7.64 | 0.417 | Superphosphate |
| Bordignon 2013d | 4 | 3.89 | 0.212 | 4 | 7.64 | 0.417 | Acidifying |
| Bordignon 2013e | 4 | 9.83 | 0.537 | 4 | 7.64 | 0.417 | Alkalizing |
| Celen and Alkis 2009a | 2 | 7.62 | 0.570 | 2 | 8.01 | 0.230 | Acidifying |
| Celen and Alkis 2009b | 2 | 8.46 | 0.530 | 2 | 8.85 | 0.460 | Acidifying |
| Chung et al. 2015 | 4 | 7.32 | 0.340 | 4 | 8.72 | 0.340 | Acidifying |
| Do et al. 2005a | 3 | 6.58 | 1.403 | 3 | 7.30 | 0.554 | Acidifying |
| Do et al. 2005b | 3 | 6.67 | 1.161 | 3 | 7.71 | 0.658 | Acidifying |
| Do et al. 2005c | 3 | 6.51 | 0.277 | 3 | 7.93 | 0.779 | Acidifying |
| Do et al. 2005d | 3 | 7.60 | 0.693 | 3 | 7.98 | 0.139 | Acidifying |
| Do et al. 2005e | 3 | 7.53 | 0.191 | 3 | 8.16 | 0.087 | Acidifying |
| Do et al. 2005f | 3 | 8.21 | 0.104 | 3 | 8.36 | 0.052 | Acidifying |
| Furlan, 2017a | 7 | 8.15 | 0.143 | 7 | 8.48 | 0.143 | Acidifying |
| Furlan, 2017b | 7 | 8.03 | 0.143 | 7 | 8.48 | 0.143 | Acidifying |
| Furlan, 2017c | 7 | 8.27 | 0.143 | 7 | 8.48 | 0.143 | Acidifying |
| Furlan, 2017d | 7 | 7.86 | 0.143 | 7 | 8.51 | 0.143 | Acidifying |
| Furlan, 2017e | 7 | 8.29 | 0.143 | 7 | 8.51 | 0.143 | Acidifying |
| Furlan, 2017f | 7 | 8.07 | 0.143 | 7 | 8.51 | 0.143 | Acidifying |
| Furlan, 2017g | 7 | 7.61 | 0.143 | 7 | 8.14 | 0.143 | Acidifying |
| Garrido et al. 2004 | 6 | 8.40 | 0.410 | 6 | 8.40 | 0.480 | Acidifying |
| Li et al., 2013 | 3 | 7.50 | 0.520 | 3 | 7.65 | 0.953 | Acidifying |
| Loch et al. 2011b | 4 | 8.62 | 0.300 | 4 | 8.89 | 0.309 | Acidifying |
| Loch et al. 2011c | 4 | 8.57 | 0.298 | 4 | 8.89 | 0.309 | Gypsum |
| Loch et al. 2011d | 4 | 9.37 | 0.326 | 4 | 8.89 | 0.309 | Alkalizing |
| Loch et al. 2011e | 4 | 9.15 | 0.318 | 4 | 8.89 | 0.309 | Alkalizing |
| Loch et al. 2011f | 4 | 9.32 | 0.324 | 4 | 8.89 | 0.309 | Adsorber |
| Loch et al. 2011g | 4 | 9.54 | 0.332 | 4 | 8.89 | 0.309 | Adsorber |
| Oliveira et al. 2003a | 4 | 7.07 | 0.308 | 4 | 8.04 | 0.351 | Acidifying |
| Oliveira et al. 2003b | 4 | 6.97 | 0.304 | 4 | 8.04 | 0.351 | Gypsum |
| Oliveira et al. 2003c | 4 | 7.85 | 0.342 | 4 | 8.04 | 0.351 | Alkalizing |
| Oliveira et al. 2004a | 4 | 7.42 | 0.501 | 4 | 8.42 | 0.568 | Acidifying |
| Oliveira et al. 2004b | 4 | 8.22 | 0.555 | 4 | 8.42 | 0.568 | Gypsum |
| Oliveira et al. 2004c | 4 | 7.80 | 0.526 | 4 | 8.42 | 0.568 | Superphosphate |
| Oliveira et al. 2004d | 4 | 9.65 | 0.651 | 4 | 8.42 | 0.568 | Alkalizing |
| Ruiz et al. 2008b | 4 | 8.38 | 0.200 | 4 | 7.65 | 0.200 | Alkalizing |
| Ruiz et al. 2008c | 4 | 8.75 | 0.200 | 4 | 7.65 | 0.200 | Alkalizing |
| Sahoo et al. 2017a | 3 | 9.10 | 0.121 | 3 | 10.3 | 0.104 | Acidifying |
| Sahoo et al. 2017b | 3 | 9.50 | 0.052 | 3 | 10.3 | 0.104 | Acidifying |
| Tasistro et al., 2007 a | 4 | 9.03 | 0.420 | 4 | 8.98 | 0.330 | Acidifying |
| Tasistro et al., 2007 b | 4 | 8.36 | 0.310 | 4 | 8.98 | 0.330 | Acidifying |
| Toppel et al., 2018a | 2 | 8.41 | 0.280 | 2 | 8.65 | 0.020 | Acidifying |
| Toppel et al., 2018b | 2 | 8.92 | 0.030 | 2 | 8.71 | 0.240 | Acidifying |
| Zhang et al., 2011a | 3 | 8.68 | 0.139 | 3 | 8.90 | 0.017 | Acidifying |
| Zhang et al., 2011b | 3 | 8.58 | 0.398 | 3 | 8.93 | 0.069 | Acidifying |
| Zhang et al., 2011c | 3 | 8.59 | 0.277 | 3 | 8.64 | 0.381 | Acidifying |
| Taherparvar et al. 2016a | 3 | 7.60 | 0.035 | 3 | 7.90 | 0.035 | Adsorber |
| Taherparvar et al. 2016b | 3 | 7.60 | 0.035 | 3 | 7.90 | 0.035 | Alkalizing |
