# Supplementary material for: An assessment of the impacts of litter treatments on the litter quality and broiler performance: A systematic review and meta-analysis
Source: PLoS One. 2020 May 6;15(5):e0232853. doi: 10.1371/journal.pone.0232853 (PMC7202646; doi:10.1371/journal.pone.0232853)
Supplement: S7 Table — (DOCX) [file pone.0232853.s007.docx]

S7_Table. Data for moisture meta-analysis

| Study name | Treated Group N | Treated Group mean | Treated Group Standard deviation | Control Group N | Control Group mean | Control Group Standard deviation | Treatment |
| --- | --- | --- | --- | --- | --- | --- | --- |
| Bordignon 2013a | 4 | 23.220 | 2.036 | 4 | 23.580 | 2.068 | Gypsum |
| Bordignon 2013b | 4 | 24.310 | 2.132 | 4 | 23.580 | 2.068 | Alkalizing |
| Bordignon 2013c | 4 | 23.850 | 2.092 | 4 | 23.580 | 2.068 | Superphosphate |
| Bordignon 2013d | 4 | 24.310 | 2.132 | 4 | 23.580 | 2.068 | Acidifying |
| Bordignon 2013e | 4 | 24.090 | 2.113 | 4 | 23.580 | 2.068 | Alkalizing |
| Do et al. 2005a | 3 | 21.060 | 5.993 | 3 | 55.070 | 1.593 | Acidifying |
| Do et al. 2005b | 3 | 43.240 | 0.987 | 3 | 49.730 | 2.286 | Acidifying |
| Do et al. 2005c | 3 | 24.700 | 8.643 | 3 | 53.730 | 1.905 | Acidifying |
| Do et al. 2005d | 3 | 42.260 | 9.301 | 3 | 51.740 | 4.018 | Acidifying |
| Do. et al. 2005e | 3 | 32.550 | 4.780 | 3 | 52.120 | 2.044 | Acidifying |
| Do. et al. 2005f | 3 | 41.000 | 6.894 | 3 | 48.240 | 3.066 | Acidifying |
| Garrido et al. 2004 | 6 | 29.600 | 38.190 | 6 | 38.300 | 13.000 | Acidifying |
| Li et al., 2013 | 3 | 31.710 | 17.753 | 3 | 31.840 | 11.172 | Acidifying |
| Loch et al. 2011b | 4 | 28.330 | 2.374 | 4 | 33.250 | 2.786 | Acidifying |
| Loch et al. 2011c | 4 | 26.440 | 2.216 | 4 | 33.250 | 2.786 | Gypsum |
| Loch et al. 2011d | 4 | 30.900 | 2.589 | 4 | 33.250 | 2.786 | Alkalizing |
| Loch et al. 2011e | 4 | 28.050 | 2.351 | 4 | 33.250 | 2.786 | Alkalizing |
| Loch et al. 2011f | 4 | 28.700 | 2.405 | 4 | 33.250 | 2.786 | Adsorber |
| Loch et al. 2011g | 4 | 26.160 | 2.192 | 4 | 33.250 | 2.786 | Adsorber |
| Nagaraj et al. 2007a | 4 | 13.000 | 3.400 | 4 | 14.000 | 3.400 | Acidifying |
| Nagaraj et al. 2007b | 4 | 13.000 | 3.400 | 4 | 14.000 | 3.400 | Acidifying |
| Nagaraj et al. 2007c | 4 | 12.000 | 3.400 | 4 | 14.000 | 3.400 | Acidifying |
| Ruiz et al. 2008b | 4 | 12.310 | 0.020 | 4 | 13.920 | 0.020 | Alkalizing |
| Ruiz et al. 2008c | 4 | 12.960 | 0.020 | 4 | 13.920 | 0.020 | Alkalizing |
| Sahoo et al. 2017a | 3 | 28.000 | 1.507 | 3 | 30.330 | 3.048 | Acidifying |
| Sahoo et al. 2017b | 3 | 26.330 | 3.256 | 3 | 30.330 | 3.048 | Acidifying |
| Zhang et al., 2011a | 3 | 40.980 | 2.806 | 3 | 38.390 | 2.563 | Acidifying |
| Zhang et al., 2011b | 3 | 54.340 | 1.264 | 3 | 48.370 | 4.936 | Acidifying |
| Zhang et al., 2011c | 3 | 57.770 | 12.384 | 3 | 56.390 | 9.977 | Acidifying |
| Taherparvar et al. 2016a | 3 | 39.410 | 6.668 | 3 | 36.410 | 6.668 | Adsorber |
| Taherparvar et al. 2016b | 3 | 39.500 | 6.668 | 3 | 36.410 | 6.668 | Alkalizing |
