# Supplementary material for: An assessment of the impacts of litter treatments on the litter quality and broiler performance: A systematic review and meta-analysis
Source: PLoS One. 2020 May 6;15(5):e0232853. doi: 10.1371/journal.pone.0232853 (PMC7202646; doi:10.1371/journal.pone.0232853)
Supplement: S8 Table — (DOCX) [file pone.0232853.s008.docx]

S8_Table. Data for pathogenic microbiota meta-analysis.

| Study name | Treated Group N | Treated Group mean | Treated Group Standard deviation | Control Group N | Control Group mean | Control Group Standard deviation | Treatment |
| --- | --- | --- | --- | --- | --- | --- | --- |
| Chung et al. 2015a | 4 | 3.37 | 0.500 | 4 | 4.36 | 0.500 | Acidifying |
| Chung et al. 2015b | 4 | 4.97 | 1.080 | 4 | 5.56 | 1.080 | Acidifying |
| Garrido et al. 2004a | 6 | 5.92 | 1.280 | 6 | 6.16 | 0.810 | Acidifying |
| Garrido et al. 2004b | 6 | 3.95 | 1.920 | 6 | 4.40 | 2.510 | Acidifying |
| Lopes et al. 2015a | 8 | 0.33 | 0.594 | 8 | 1.75 | 0.198 | Alkalizing |
| Lopes et al. 2015d | 8 | 1.49 | 0.481 | 8 | 2.41 | 0.057 | Alkalizing |
| Lopes et al. 2015g | 8 | 0.36 | 0.396 | 8 | 1.64 | 0.707 | Alkalizing |
| Sahoo et al. 2017a | 3 | 8.42 | 0.087 | 3 | 8.64 | 0.069 | Acidifying |
| Sahoo et al. 2017b | 3 | 8.50 | 0.139 | 3 | 8.64 | 0.069 | Acidifying |
| Sahoo et al. 2017c | 3 | 7.78 | 0.294 | 3 | 8.16 | 0.121 | Acidifying |
| Sahoo et al. 2017d | 3 | 7.92 | 0.069 | 3 | 8.16 | 0.121 | Acidifying |
| Sahoo et al. 2017e | 3 | 8.78 | 0.052 | 3 | 9.05 | 0.104 | Acidifying |
| Sahoo et al. 2017f | 3 | 8.65 | 0.052 | 3 | 9.05 | 0.104 | Acidifying |
| Sampaio et al. 1999a | 4 | 9.47 | 0.014 | 4 | 9.46 | 0.014 | Gypsum |
| Sampaio et al. 1999b | 4 | 9.44 | 0.014 | 4 | 9.46 | 0.014 | Gypsum |
| Sampaio et al. 1999c | 4 | 9.25 | 0.014 | 4 | 9.46 | 0.014 | Gypsum |
| Sampaio et al. 1999d | 4 | 8.30 | 0.012 | 4 | 9.46 | 0.014 | Gypsum |
| Taherparvar et al. 2016a | 3 | 10.05 | 0.225 | 3 | 10.34 | 0.225 | Adsorber |
| Taherparvar et al. 2016b | 3 | 10.16 | 0.225 | 3 | 10.34 | 0.225 | Alkalizing |
| Taherparvar et al. 2016c | 3 | 9.56 | 0.173 | 3 | 9.82 | 0.173 | Adsorber |
| Taherparvar et al. 2016d | 3 | 9.80 | 0.173 | 3 | 9.82 | 0.173 | Alkalizing |
